# Incrustations in Soil-Pipes

**Published:** 1920-11-27

**Authors:** 


### INCRUSTATIONS IN SOIL-PIPES.

In these days when so much importance is attached to the question of public health it seems extraordinary that so little attention is bestowed on the cleanliness and appearance of the water-closet pans. On inspection it is apparent that these pans, unless they receive thorough daily cleansing, become corroded to a varying degree. These incrustations occur in two situations. (1) In the S-shaped portion of the trap, where they mostly form; (2) in the descending portion leading to the soil-pipe; here the amount is comparatively slight.

These urinary deposits consist of carbonates, phosphates, and sulphates of calcium, which after a process of time form large accumulations, so that the pipe instead of having a diameter of 4 inches, is often reduced to 1 inch, and it is only a question of time before it becomes finally blocked. The weight of these deposits frequently exceed 2¾ lb. in the S part, and 1¾ lb. in the descending part, sometimes measuring as much as 16 inches in length.

By reason of the calcium sulphate in the incrustations the deposit becomes intensely hard and no acid will dissolve it. With careful scraping the pans can be made quite clean, but the operation requires a man skilled in the work, otherwise he will break them. Special but simple tools have to be made in order to reach round the bend of the trap. These implements can be easily made by an engineer, and a man in the Guardians' employ, with suitable tools, has cleaned and scraped the whole of the 263 pans in an infirmary and institution without a single breakage.

To obviate the formation of incrustations the crown of the trap should be fitted with a detachable cap which can easily be removed to enable the parts where the deposits occur to be periodically scraped and cleaned. The cap would have to be fitted in such a way as to render it impossible to allow sewer gas to escape into the building. By such a device the expenditure, which at present is heavy for supplying and fitting new pans, would be minimised.

In order to keep pans clean they should be thoroughly cleansed with a brush and a pail of hot, soapy water once a day. Once a month the pans should be emptied of water, and some strong hydrochloric acid allowed to stand in the bottom of the trap for a few minutes, then finally flushed with water. If the pans were kept clean in this way it is undoubted that there would be a great improvement in the health of the institution or house, and a great economy would be effected in public buildings in the use of disinfectants, which certainly have a pleasant odour, but which assist still further to corrode the traps by reason of their stickiness.

